# Comparative Analysis of Breast Cancer Metabolomes Highlights Fascin’s Central Role in Regulating Key Pathways Related to Disease Progression

**DOI:** 10.3390/ijms25147891

**Published:** 2024-07-18

**Authors:** Reem H. AlMalki, Huda K. Al-Nasrallah, Alanoud Aldossry, Rayanah Barnawi, Samiyah Al-Khaldi, Sheema Almozyan, Mysoon M. Al-Ansari, Hazem Ghebeh, Anas M. Abdel Rahman, Monther Al-Alwan

**Affiliations:** 1 Metabolomics Section, Department of Clinical Genomics, Center for Genomics Medicine, King Faisal Specialist Hospital and Research Centre, Riyadh 11211, Saudi Arabia; rgalmalki@kfshrc.edu.sa; 2 Cell Therapy and Immunobiology Department, King Faisal Specialist Hospital and Research Centre, Riyadh 11211, Saudi Arabia; halnasrallah@kfshrc.edu.sa (H.K.A.-N.); aaldossry@kfshrc.edu.sa (A.A.); barnawir@ssa.gov.sa (R.B.); salkhaldi@kacst.edu.sa (S.A.-K.); salmozyan@kfshrc.edu.sa (S.A.); hghebeh@kfshrc.edu.sa (H.G.); 3 Applied Genomics Technologies Institute, Health Sector, King Abdulaziz City for Sciences and Technology, Riyadh 11442, Saudi Arabia; 4 Department of Molecular Oncology, Cancer Biology & Experimental Therapeutics Section, King Faisal Specialist Hospital and Research Centre, Riyadh 11211, Saudi Arabia; myalansari@ksu.edu.sa; 5 Department of Botany and Microbiology, College of Science, King Saud University, Riyadh 11451, Saudi Arabia; 6 College of Medicine, Al-Faisal University, Riyadh 11533, Saudi Arabia

**Keywords:** breast cancer, metabolic pathways, fascin, untargeted metabolomics

## Abstract

Omics technologies provide useful tools for the identification of novel biomarkers in many diseases, including breast cancer, which is the most diagnosed cancer in women worldwide. We and others have reported a central role for the actin-bundling protein (fascin) in regulating breast cancer disease progression at different levels. However, whether fascin expression promotes metabolic molecules that could predict disease progression has not been fully elucidated. Here, fascin expression was manipulated via knockdown (fascin^KD+NORF^) and rescue (fascin^KD+FORF^) in the naturally fascin-positive (fascin^pos+NORF^) MDA-MB-231 breast cancer cells. Whether fascin dysregulates metabolic profiles that are associated with disease progression was assessed using untargeted metabolomics analyses via liquid chromatography–mass spectrometry. Overall, 12,226 metabolic features were detected in the tested cell pellets. Fascin^pos+NORF^ cell pellets showed 2510 and 3804 significantly dysregulated metabolites compared to their fascin^KD+NORF^ counterparts. Fascin rescue (fascin^KD+FORF^) revealed 2710 significantly dysregulated cellular metabolites compared to fascin^KD+NORF^ counterparts. A total of 101 overlapped cellular metabolites between fascin^KD+FORF^ and fascin^pos+NORF^ were significantly dysregulated in the fascin^KD+NORF^ cells. Analysis of the significantly dysregulated metabolites by fascin expression revealed their involvement in the metabolism of sphingolipid, phenylalanine, tyrosine, and tryptophan biosynthesis, and pantothenate and CoA biosynthesis, which are critical pathways for breast cancer progression. Our findings of fascin-mediated alteration of metabolic pathways could be used as putative poor prognostic biomarkers and highlight other underlying mechanisms of fascin contribution to breast cancer progression.

## 1. Introduction

Breast cancer is the most common malignancy in women worldwide. It can be classified into four major subtypes based on the expression of estrogen receptor (ER), progesterone receptor (PR), human epidermal growth factor receptor 2 (HER2), and Ki67 index [1]. These four major subtypes are Luminal A (ER+ and/or PR+, HER2−, Ki-67 < 14%), luminal B (ER+ and/or PR+, HER2−, Ki-67 ≥ 14%; or ER+ and/or PR+, HER2+), HER2-enriched (ER−, PR−, HER2+), and triple negative breast cancer (TNBC; ER−, PR−, HER2−). While there has been substantial progress in the development of targeted therapeutics for breast cancer, tumor-related mortality has remained high, especially in TNBC, due to limited therapeutic options, drug resistance, and metastasis [2]. While it accounts for around 15% of breast cancer cases, TNBC is considered to be the most aggressive subtype with the worst outcome [3]. Therefore, a better understanding of the biology of TNBC could open a new window for therapeutic intervention that could halt the disease progression and improve survival outcomes.

Metabolomics is a rapidly growing field in modern life science research, which focuses on endogenous low-molecular-weight molecules referred to as metabolites [4,5]. The metabolomes comprise several constituents, including amino acids, fatty acids, carbohydrates, vitamins, and lipids, which could reveal changes in biochemical reactions and their associated pathways within the living cells [6]. Analysis of the metabolomes is a very useful approach to determine dynamic changes within living cells and could reflect biological responses to biochemical reactions [7]. Furthermore, metabolites provide valuable knowledge that could be used to differentiate between normal and diseased cells, better understand the link between dysregulated metabolisms and disease progression, or define the underlying mechanism of the disease, or they could be used as potential targets for therapeutic intervention [8,9]. In breast cancer, metabolites such as glutamate-to-glutamine ratio and aerobic glycolysis have been used to differentiate between ER and HER2 molecular subtypes [10]. Furthermore, metabolites such as glycolysis, TCA cycle, and beta-oxidation were shown to predict tumor aggressiveness, where they were reported to be higher in TNBC than in hormone receptor-positive breast cancer cases [11,12,13]. Therefore, metabolomics screening can provide an early sign that can be used to predict the aggressiveness and response of a breast cancer patient to a specific treatment.

We have previously demonstrated the expression of an actin-bundling protein (fascin) in a high percentage of TNBC, which was significantly associated with metastasis [14] and drug resistance [15]. These key factors promote disease progression and poor survival. Furthermore, comprehensive transcriptome and pathway analyses revealed a critical role for fascin in promoting TNBC progression [16]. Previously, fascin has been reported to exert resistance to metabolic stress, such as glucose limitation or biguanide treatment [17]; however, its effect on the metabolism of TNBC has not been well characterized. This study assessed the effect of fascin expression on the metabolomics profile of the MDA-MB-231 breast cancer cell line, a model of TNBC. Both cell pellets and their secretomes showed significantly dysregulated metabolites in fascin-positive cells compared to their fascin knockdown counterparts. Fascin expression dysregulated metabolic pathways that were reported to be critical for breast cancer progression. These findings could help expand our understanding of how fascin regulation of specific metabolites could promote breast cancer progression and may open a new window for therapeutic intervention via targeting fascin or its dysregulated metabolites.

## 2. Results

### 2.1. Fascin Expression Regulates Functions of MDA-MB-231 Cells Linked to Breast Cancer Progression

**Figure 1:**
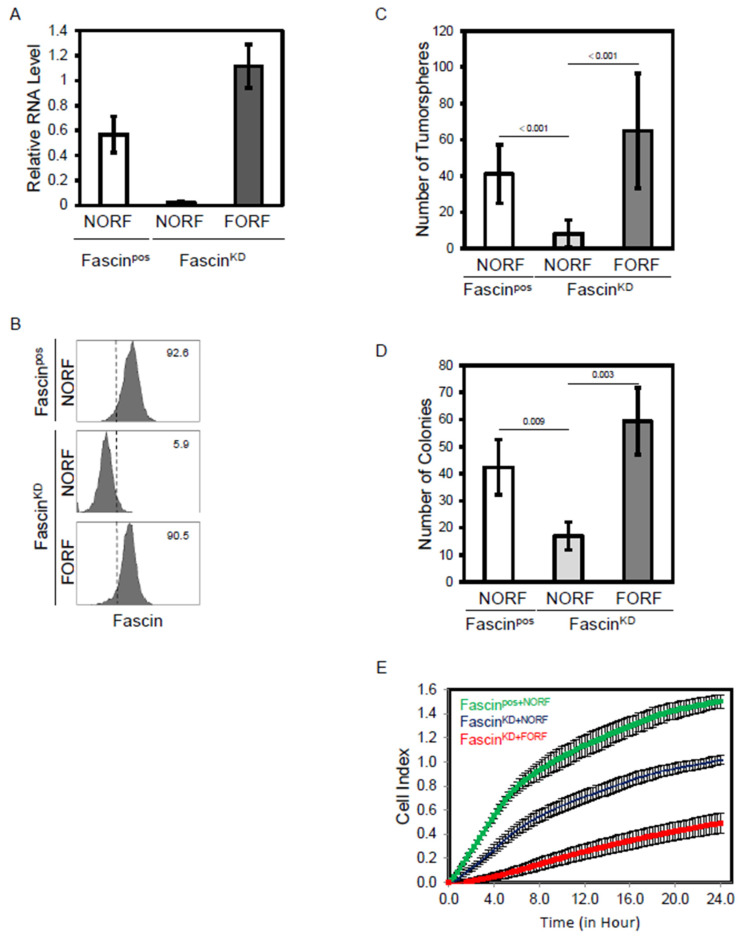
(**A**–**E**): Fascin knockdown reduced tumorsphere, colony forming ability, and migration of MDA-MB-231 cells. (**A**) Bar graph showing the relative RNA expression of fascin in MDA-MB-231 cells following its manipulation (knockdown and rescue). (**B**) FACS histograms showing fascin protein expression following its manipulation. The groups are as described in the methods: the naturally fascin positive + empty ORF (fascin^pos+NORF^), fascin knockdown + empty ORF (fascin^KD+NORF^) and fascin rescued in the knockdown + fascin ORF (fascin^KD+FORF^). The numbers displayed on the histograms are the percentage of fascin positive cells in reference to the isotype control (dotted lines). Bar graph showing the number of tumorspheres (**C**) and colonies (**D**) formed by MDA-MB-231 cells following fascin manipulation. (**E**) Live cell assays showing migration of MDA-MB-231 cells following fascin manipulation. Results are the mean of triplicates ± SD and are representative of three independent experiments.

We have previously reported a critical role for fascin in regulating the disease progression of triple-negative breast cancer (TNBC). However, the effect of fascin on the metabolomics profile of TNBC has not been previously elucidated. The MDA-MB-231 cell line is a widely used TNBC model expressing a high level of fascin [14,18]. Thus, knockdown and rescue experiments were used to study the effect of its expression on the metabolomics profile in this breast cancer model. We have previously generated stable fascin knockdown (fascin^KD^) in MDA-MB-231 cells using specific shRNA, and its expression was then rescued using fascin ORF (fascin^KD+FORF^) or empty ORF (fascin^KD+NORF^) as a control [19]. The fascin+ group, generated using scrambled ShRNA, was also subjected to empty ORF (fascin^pos+NORF^) as a transfection control. Fascin knockdown and rescue were confirmed at the RNA levels using RT-qPCR (Figure 1A) and at the protein levels using flow cytometry (Figure 1B). The effect of fascin knockdown and rescue in breast cancer cells has also been tested using epithelial-to-mesenchymal transition (EMT) markers as well as stemness functional assays, which are known to regulate disease progression. Decreased expression of the mesenchymal marker (vimentin) and increased expression of the epithelial marker (E-cadherin) were observed in fascin^KD+NORF^ compared to their fascin^pos+NORF^ counterparts (Figure S1A,B). These mesenchymal and epithelial markers were reversed when fascin expression was rescued in fascin knockdown cells (fascin^KD+FORF^). Functionally, the decreased tumorsphere formation ability by the knockdown group (fascin^KD+NORF^) was restored when fascin expression was rescued (fascin^KD+FORF^) to comparable levels of their fascin expressing (fascin^pos+FORF^) counterparts (Figure 1C). Similarly, rescuing fascin expression (fascin^KD+FORF^) restored their colony-forming ability (Figure 1D) and migration (Figure 1E) to levels comparable to that of their fascin-expressing (fascin^pos+FORF^) counterparts. Altogether, these data established the fascin loss and gain of function model and linked it to key functions known to regulate breast cancer progression.

### 2.2. Fascin Expression Dysregulates the Metabolomics Profile of MDA-MB-231 Cells

The effect of fascin on the progression of breast cancer has been established by many research teams, including our group (reviewed in [20]). Toward understanding fascin’s role in this process, we dived deeper to assess its effect on the metabolomics profile of breast cancer. Cells and secretomes were collected from the knockdown (fascin^KD+NORF^) and rescued (fascin^KD+FORF^) cells, as well as from the fascin-expressing control (fascin^pos+FORF^) group. Overall, 14,874 mass ion features were detected in both the positive (11,966) and negative (2908) ionization modes (Table S1). The data were deposited in Metabolomics Workbench (ST003305).

#### 2.2.1. A-Dysregulated Metabolomics Profile and Pathway Analysis of MDA-MB-231 Cell Pellet by Fascin Expression

**Figure 2:**
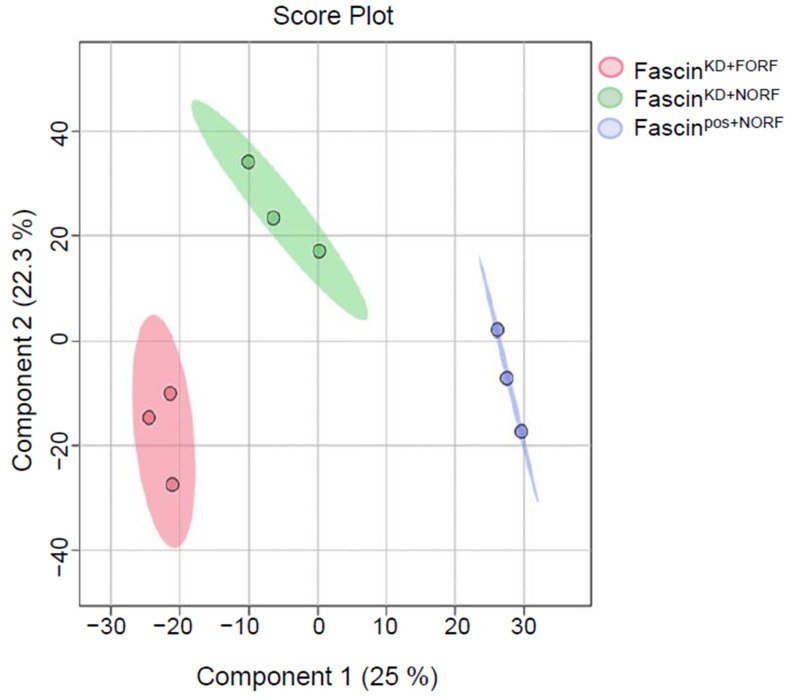
PLS-DA showed clear separation in MDA-MB-231 cell pellets following fascin knockdown and restoration. PLS-DA analysis of cell pellets showing separation between fascin^pos+NORF^, fascin^KD+NORF^, and fascin^KD+FORF^ groups.

**Figure 3:**
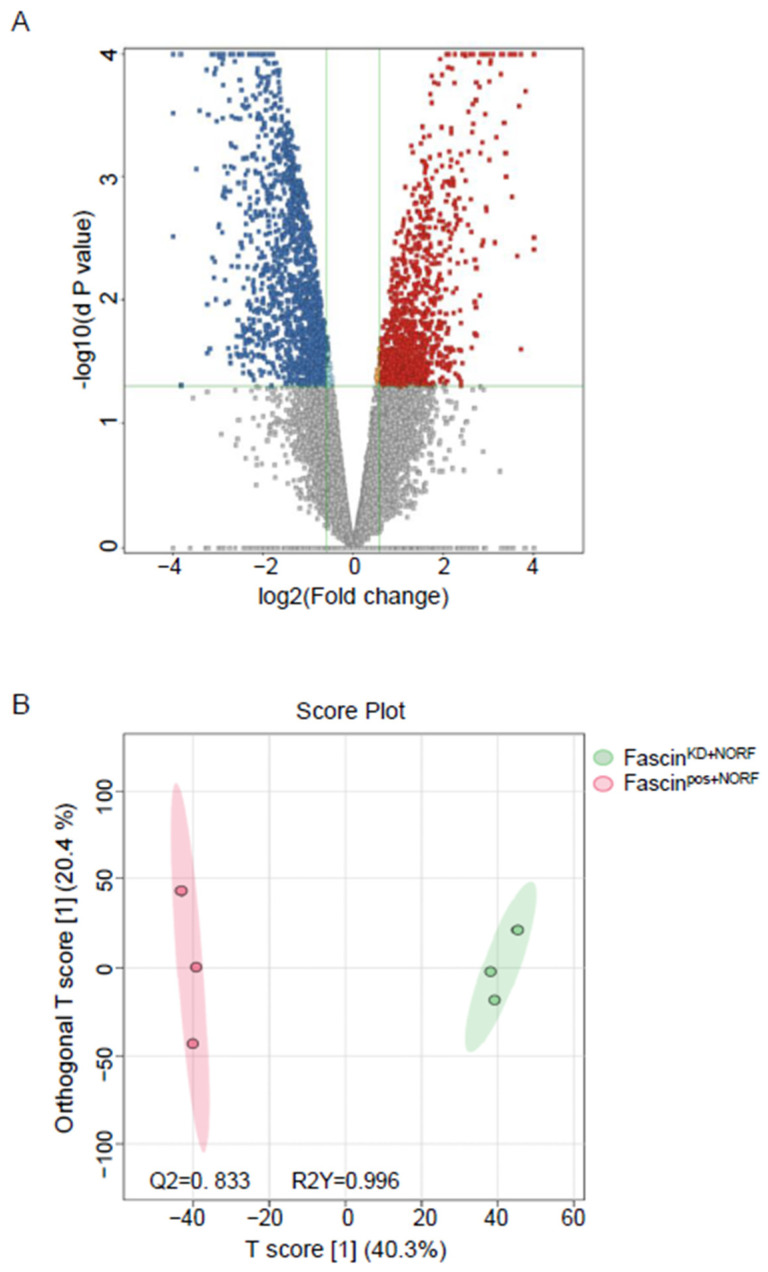
(**A**,**B**): Dysregulated metabolites in MDA-MB-231 cell pellets following fascin knockdown. (**A**) Volcano plot showing the 2510 significantly dysregulated metabolites between fascin^pos+NORF^ and fascin^KD+NORF^ cells, with 1029 and 1481 metabolites up- (red) and downregulated (blue), respectively. (**B**) OPLS-DA model shows evident separation between fascin^pos+NORF^ and fascin^KD+NORF^ cells. The robustness of the created models was evaluated by the fitness of model (R2Y = 0.996) and predictive ability (Q2 = 0.833) values in a larger dataset (n = 100).

**Figure 4:**
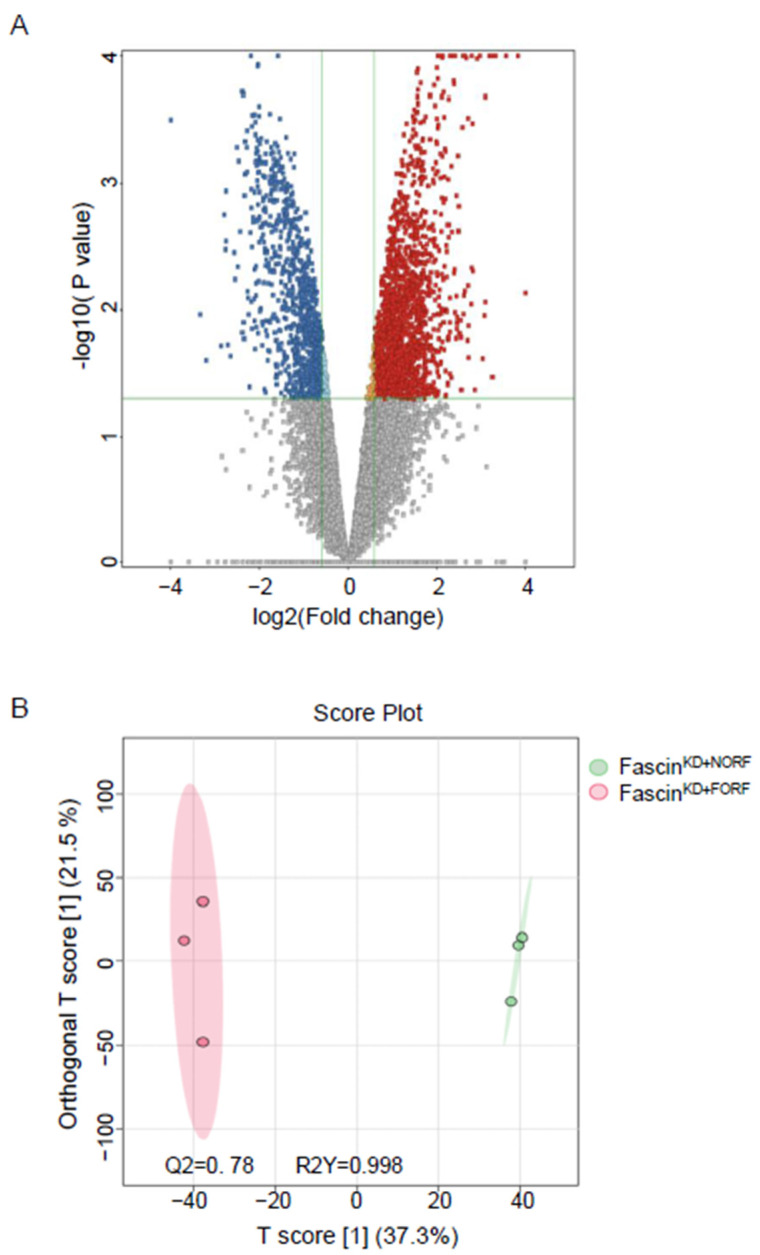
(**A**,**B**): Dysregulated metabolites in MDA-MB-231 cell pellets following fascin restoration in the knockdown cells. (**A**) Volcano plot showing the 2710 significantly dysregulated metabolites between fascin^KD+NORF^ and fascin^KD+FORF^ cells, with 1804 and 906 metabolites up- (red) and downregulated (blue), respectively. (**B**) OPLS-DA model shows evident separation between fascin^KD+NORF^ and fascin^KD+FORF^ cells. The robustness of the created models was evaluated by the fitness of model (R2Y = 0.998) and predictive ability (Q2 = 0.78) values in a larger dataset (n = 100).

**Figure 5:**
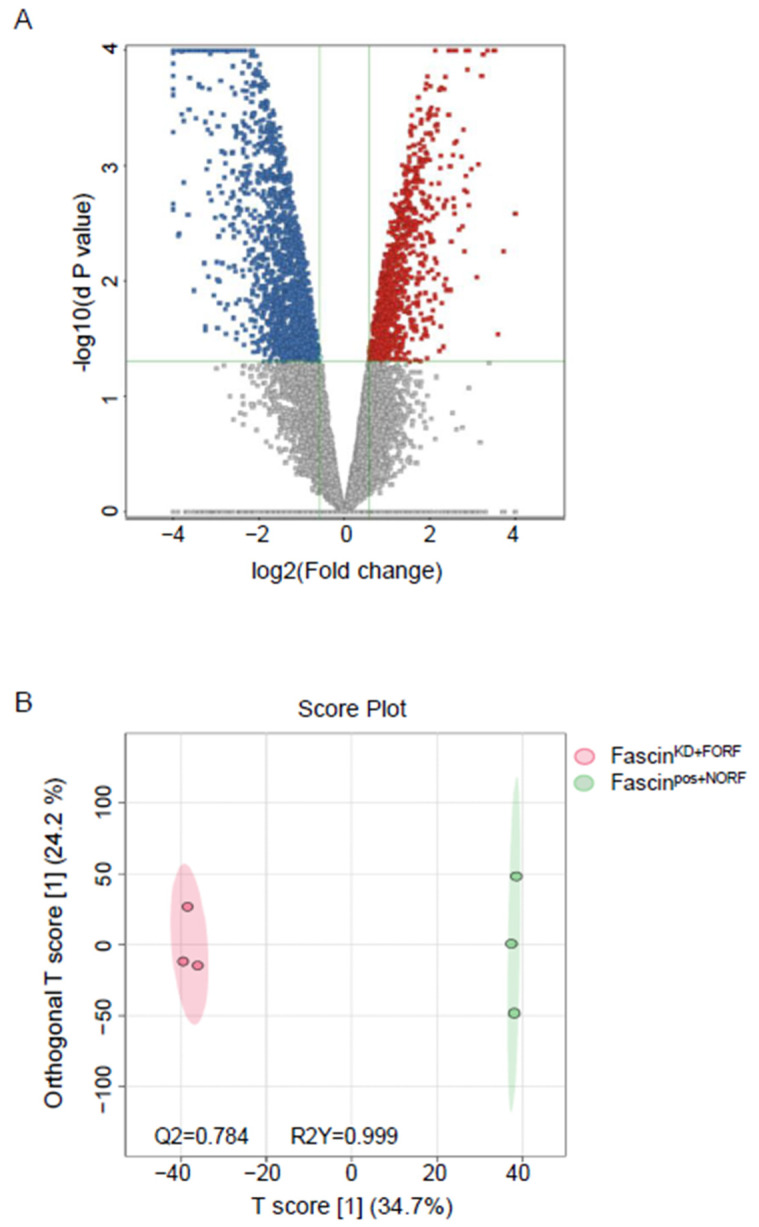
(**A**,**B**): Dysregulated metabolites in MDA-MB-231 cell pellets between fascin^pos+NORF^ and fascin^KD+FORF^ cells. (**A**) Volcano plot showing 2562 significantly dysregulated metabolites between fascin^pos+NORF^ and fascin^KD+FORF^ cells, with 1008 and 1554 metabolites up- (red) and downregulated (blue), respectively. (**B**) OPLS-DA model shows evident separation between fascin^pos+NORF^ and fascin^KD+FORF^ cells. The robustness of the created models was evaluated by the fitness of model (R2Y = 0.999) and predictive ability (Q2 = 0.784) values in a larger dataset (n = 100).

The detected compound ions were evaluated using partial least squares–discriminant analysis (PLS-DA), a multivariate analysis method. This analysis showed replicates clustering and clear separation between the three study groups in the cell pellets (Figure 2). The cell pellet features were univariately analyzed using Mass Profiler Professional (MPP) software v15.0 after filtering them by frequency (cut-off percentage 80 of all samples). Only 12,226 features were statistically evaluated using a moderated *t*-test *p* < 0.05, and fold change cut-off of 1.5. There were significantly dysregulated metabolites (n = 2510) between fascin knockdown cells (fascin^KD+NORF^) and their fascin-expressing counterparts (fascin^pos+NORF^), where 1029 and 1481 metabolites were up- and downregulated, respectively (Figure 3A and Table S2A). However, only 272 were identified as human endogenous metabolites (Table S2B). The OPLS-DA prediction model, based on 14,874 metabolites/features, showed a clear separation between the fascin^KD+NORF^ and fascin^pos+NORF^ cells with very high computed predictive ability and fitness values of Q2: 0.833 and R2Y: 0.996, respectively (Figure 3B). Similarly, there were 2710 significantly dysregulated metabolites between fascin^KD+NORF^ cells and their rescued (fascin^KD+FORF^) counterparts, with 1804 and 906 metabolites being up- and downregulated, respectively (Figure 4A and Table S3A). However, only 448 were identified as human endogenous metabolites (Table S3B). The OPLS-DA model separated the fascin^KD+NORF^ and fascin^KD+FORF^ with very high computed predictive ability and fitness values of Q2 = 0.78 and R2Y = 0.998 (Figure 4B). Interestingly, there were 2562 significantly dysregulated metabolites between the fascin knockdown rescued cells (fascin^KD+FORF^) and their fascin-expressing counterparts (fascin^pos+NORF^), with 1008 and 1554 metabolites up- and downregulated, respectively (Figure 5A and Table S4A). However, only 288 were identified as human endogenous metabolites (Table S4B). The OPLS-DA model separated the fascin^KD+FORF^ and fascin^pos+NORF^ cells with high computed predictive ability and fitness values of Q2 = 0.784 and R2Y = 0.999, respectively (Figure 5B).

Among the annotated compounds (2639) in cell pellets, 623 metabolites were identified as human endogenous metabolites (Table S5). The Venn diagram showed 272 significantly dysregulated metabolites related to fascin^KD+NORF^ (Figure S2). Importantly, 101 out of the 272 dysregulated metabolites in the cell pellets of fascin^KD+NORF^ compared to fascin^pos+NORF^ were restored when fascin expression was rescued in the fascin^KD+FORF^ group (Figure S2 and Table S6).

**Figure 6:**
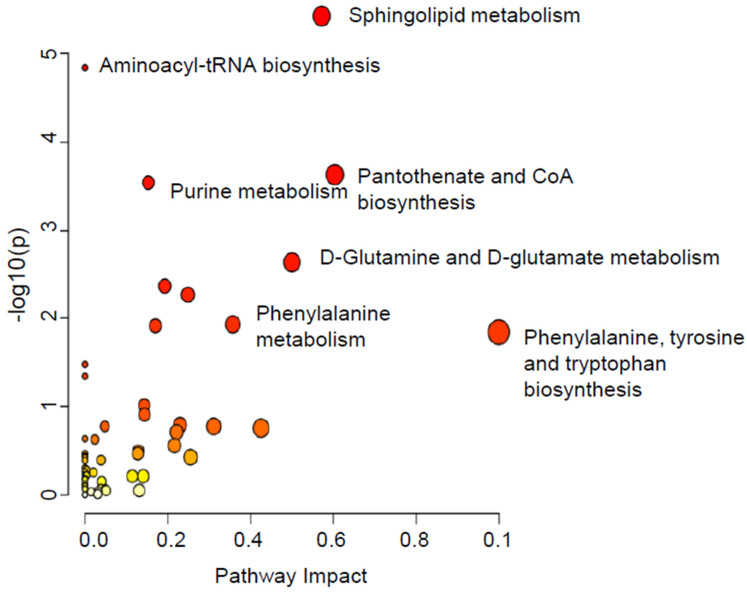
Pathway analysis of the significantly dysregulated metabolites in MDA-MB-231 cell pellets following fascin manipulation. Variation in colors (yellow to red) indicates increase in the significance level of the dysregulated metabolites.

To gain insight into the effect of fascin-mediated dysregulation of metabolites on the different pathways, the pathway analysis of the significantly dysregulated metabolites between the three groups was conducted, and it revealed 35 significant metabolic pathways in the cell pellets (Figure 6). The most affected pathways were phenylalanine, tyrosine, and tryptophan biosynthesis, sphingolipid metabolism, pantothenate, and CoA biosynthesis, and D-glutamine and D-glutamate metabolism (Figure 6).

#### 2.2.2. B-Dysregulated Metabolomics Profile and Pathway Analysis of MDA-MB-231 Secretomes by Fascin Expression

The results above showed a role for fascin in regulating metabolites in MDA-MB-231 breast cancer cells. Here, we tested if the metabolic profile of MDA-MB-231 secretomes is also altered by fascin expression. Similarly, all detected (11,770) ions in secretomes. Partial least squares–discriminant analysis (PLS-DA) showed sample clustering and clear separation between the three tested groups in the secretomes (Figure S3). The binary comparisons were evaluated using a moderated *t*-test *p* < 0.05, FC 1.5. This analysis revealed 3804 significantly dysregulated metabolites between fascin^KD+NORF^ and fascin^pos+NORF^, with 1397 and 2407 metabolites being up- and downregulated, respectively (Figure S4A and Table S7A). Only 528 were identified as human endogenous metabolites (Table S7B). The OPLS-DA model showed a clear separation between the fascin^KD+NORF^ and fascin^pos+NORF^ groups with very high computed predictive ability and fitness values of Q2: 0.898 and R2Y: 0.999 (Figure S4B). There were 452 significantly dysregulated metabolites between the fascin^KD+NORF^ and fascin^KD+FORF^ groups, with 205 and 247 metabolites up- and downregulated, respectively (Figure S5A and Table S8A). Only 49 were identified as human endogenous metabolites (Table S8B). The OPLS-DA model showed a clear separation between the fascin^KD+NORF^ and fascin^KD+FORF^ groups with very high computed predictive ability and fitness values of Q2 = 0.498 and R2Y = 0.997 (Figure S5B). Interestingly, there were 802 significantly dysregulated metabolites between the fascin^KD+FORF^ and fascin^pos+NORF^ groups, with 252 and 550 metabolites being up- and downregulated, respectively (Figure S6A and Table S9A). However, only 133 were identified as human endogenous metabolites (Table S9B). The OPLS-DA model separated the fascin^KD+FORF^ and fascin^pos+NORF^ with high computed predictive ability and fitness values of Q2 = 0.733 and R2Y = 0.999 (Figure S6B). 

**Figure 7:**
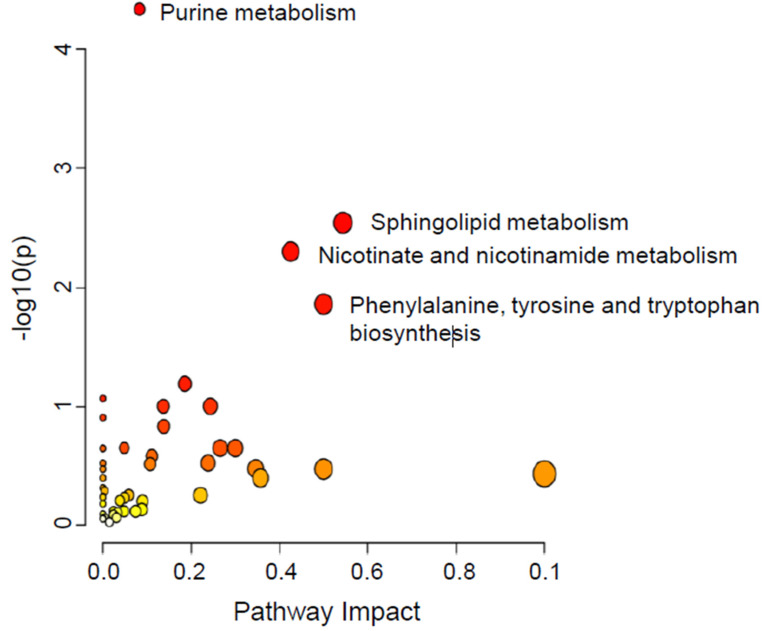
Pathway analysis for the significantly dysregulated metabolites in MDA-MB-231 secretomes following fascin manipulation. Variation in colors (yellow to red) indicates increase in the significance level of the dysregulated metabolites.

Out of all annotated compounds (3215) in cell secretomes, only 594 metabolites were identified as human endogenous metabolites (Table S10). The Venn diagram showed 528 significantly dysregulated metabolites related to fascin^KD+NORF^ (Figure S7). Similar to the cell pellets, 16 of the 528 dysregulated metabolites in the secretomes of fascin^KD+NORF^ compared to fascin^pos+NORF^ were restored when fascin expression was rescued in the fascin^KD+FORF^ group (Figure S7 and Table S11). Interestingly, 230 significantly dysregulated metabolites existed between cell pellets and their secretomes (Figure S8 and Table S12). The pathway analysis of the considerably dysregulated metabolites between the three groups revealed that the impacted pathways were sphingolipid metabolism, phenylalanine, tyrosine, and tryptophan biosynthesis, and nicotinate and nicotinamide metabolism (Figure 7).

Interestingly, there were 27 overlapping dysregulated pathways between cell pellets and their secretomes. Among these pathways were phenylalanine, tyrosine, tryptophan biosynthesis, pantothenate, and CoA biosynthesis (Figure S9). Altogether, these data from knockdown and rescued experiments strongly support the idea that the observed dysregulation in the metabolic profile was more likely fascin-dependent and not due to an off-target effect, and reveal a central role for fascin in modulating key metabolic pathways, which were reported to regulate the progression of various tumors, including breast cancer.

## 3. Discussion

Metabolomics analyses have recently gained much attention by providing immense information linked to disease diagnosis, progression, or response to therapy. Furthermore, identifying a unique metabolite signature can be used to detect or continuously monitor dynamic changes during disease development and progression. This can be particularly important in the case of cancer, as early detection and prediction of the drug efficacy can have a major impact on the patient outcome. The findings reported in this study show that fascin modulates the metabolites in breast cancer cells and their secretomes, extends knowledge, and contributes to the evolving understanding of fascin’s contribution to promoting breast cancer progression.

Fascin expression in various types of cancer, including breast cancer, has always been linked to poor prognosis, aggressive behavior, and shorter survival [21,22,23,24]. Different signal transduction pathways that regulate cell growth, migration, invasion, and drug resistance were found to be under the control of fascin. However, very little information is available on whether fascin expression in breast cancer modulates their metabolites. Under metabolic stress, fascin is recruited to the mitochondria to stabilize actin filaments and confer resistance, promoting lung cancer metastatic colonization [17]. Metabolic profiling has been shown to provide useful information in predicting disease progression. For example, Arnone AA et al. showed an association between endogenous estrogens and changes in breast metabolic signaling, especially those related to glucose and fatty acid metabolism [25]. Metabolites such as glycolysis have not only been used to differentiate between ER and HER2 molecular subtypes [10] but also can predict the aggressive behavior of breast cancer [11,12]. Indeed, a high expression level of the glycolytic enzyme GLUT-1, a glucose transporter, was reported in TNBC, and its selective inhibitor (BAY-876) constrains their growth [26]. Furthermore, assessment of secreted deconjugated hormones and their metabolites in the biliary drainage of patients treated with tamoxifen has been used to predict hormone therapy resistance and the aggressive behavior of the tumor [27].

Our comprehensive metabolomics analysis of fascin-manipulated breast cancer cells revealed several altered metabolites involved in the sphingolipid metabolism, phenylalanine, tyrosine, and tryptophan biosynthesis, and pantothenate and CoA biosynthesis. The finding that fascin expression dysregulates sphingolipid metabolism is consistent with the reported role of this pathway in regulating cancer progression. Hydrolysis of ceramide, a sphingolipid metabolite, yields sphingosine, which in turn is phosphorylated by sphingosine kinase 1 or 2 (SPHK1 or SPHK2) to generate sphingosine 1-phosphate (S1P), which signals via S1P receptors (S1PR) (reviewed in [28]). Alternatively, ceramides can also be produced in a de novo synthesis pathway due to 3-dehydrosphinganine reduction to form sphingosine, which is acylated to produce dihydroceramides. Our study showed reduced sphingosine, 3-dehydrosphinganine, and sphinganine in fascin knockdown cells. Ceramide has been reported to activate the NF-κB signaling pathway [29], which, in combination with STAT3, was demonstrated to promote fascin expression in metastatic breast cancer [30]. Moreover, TNBC patients with increased SPHK1/NF-κB/FSCN1 axis activation showed more metastasis and poor clinical outcomes. Importantly, tumor growth and lung metastasis were suppressed when SPHK1 and NF-κB were therapeutically inhibited in the orthotopic syngeneic TNBC mouse model [31]. In agreement with the reduced levels of sphingolipid metabolites upon fascin knockdown in the current study, we have previously shown suppressed activation of the NF-κB signaling pathway and decreased migration and invasion of fascin knockdown breast cancer cells [14]. Another group showed that treating breast cancer cells with doxorubicin liberates ceramide at the plasma membrane to enhance migration and invasion [32]. In prostate and bladder cancer cells, systemic SPHK1/S1P signal through SiPR2 promotes lung metastasis via repressing the breast cancer metastasis suppressor 1 (BRMS1) [33]. Interestingly, we had previously demonstrated an inverse relationship between fascin and nuclear BRMS1 in breast cancer patients. We confirmed this observation in vitro, where BRMS1 increased when fascin was knocked down in the TNBC cell line [14]. These data suggest a crosstalk between sphingolipid and fascin in promoting breast cancer progression.

Our fascin knockout breast cancer cells show increased pyridoxal phosphate, the active form of vitamin B6, which has been reported to catalyze many enzymatic reactions and to suppress cell proliferation in melanoma and colon cancer [34,35]. However, results from previous studies that investigated the link between dietary vitamin B6 and breast cancer risk were inconclusive. For example, Lurie et al. showed a correlation between a higher circulating level of vitamin B6 and a reduced risk of postmenopausal breast cancer [36]. However, Zhang et al. found no link between the level of vitamin B6 and breast cancer risk, at least in the younger women group [37]. The increased level of methylthioadenosine in our fascin knockdown model is consistent with the reported function of this metabolite as a tumor suppressor in breast cancer [38]. Indeed, methylthioadenosine phosphorylase, the main enzyme that maintains methylthioadenosine at low levels, was abundantly expressed in normal human tissues and cells [39]. On the contrary, frequent deletion of the methylthioadenosine phosphorylase gene was reported in many tumor-derived cell lines and types of cancer, including breast [38]. In the human osteosarcoma cell line, high expression of gangliosides GD3 and GD2 was reported to correlate with increased phosphorylation of breast cancer anti-estrogen resistance protein 1, paxillin, and focal adhesion kinase (FAK) [40]. Furthermore, ganglioside GD3 recruits beta 1 integrin into lipid rafts and enhances the adhesion of melanoma cells [41]. The findings that GD3 mediated increased phosphorylation of FAK and recruitment of beta 1 integrin into lipid rafts to regulate cell adhesion and other malignancy features suggest a crosstalk between GD3 and fascin. The reduced level of GD3 in our fascin knockdown cells is consistent with our previous studies, where we demonstrated a fascin-mediated effect on both beta 1 integrin expression [19] and FAK activation [15] in breast cancer cells. The link between fascin and these metabolites may explain some of the reported functions for this protein in breast cancer.

Tryptophan biosynthesis is one of the key metabolic pathways for protein synthesis that can be catalyzed by different enzymes, including indoleamine 2,3 dioxygenase 1 (IDO1), indoleamine 2,3 dioxygenase 2 (IDO2), and tryptophan 2,3 dioxygenase (TDO2), to generate several metabolites in the kynurenine pathway (reviewed in [42]). Previous studies reported higher expression of IDO1 in several human cancers, which correlates with poor prognosis [43]. In a mouse model (MMTV/neu) of spontaneous mammary cancer, loss of the tumor suppressor (Bin1) increased IDO expression to support a more aggressive tumor [44]. Importantly, treatment of these mammary tumors with 1-MT, an IDO inhibitor, potentiates the cytotoxic effect of paclitaxel, as demonstrated by the significant regression in the tumor size. In another study, Heng B et al. showed a correlation between high expression of IDO1 in breast cancer with poor outcome, increased infiltration of Foxp3+ regulatory T cells, and lymph node metastasis [42]. The ability of IDO inhibitors to reverse immune evasion further confirmed the role of tryptophan metabolism in promoting immune escape and distant metastasis. The findings that fascin regulates few tryptophan metabolites may explain how fascin contributes to the disease progression. Our study found that fascin knockdown upregulates some metabolites, such as L-cysteine, which is known to contribute to the development and progression of cancer via different mechanisms, including post-translational modification [45]. This goes against the established role of fascin in promoting breast cancer progression and chemoresistance. Furthermore, fascin knockdown in our breast cancer cells correlates with an increased level of phenylalanine, which is known to be elevated in the plasma of ovarian cancer patients and has been linked to immune activation and inflammation [46]. Future studies should investigate whether elevated phenylalanine levels in our fascin knockdown cells correlate with better immune activation. 

While this study demonstrated a role for fascin in regulating metabolic profile in TNBC, it has several limitations. The conclusion was based on fascin knockdown and rescue experiments in one TNBC cell line. Future studies should consider using other approaches of fascin manipulation such as the small molecule fascin inhibitor (NP-G2-044) or using different fascin ShRNAs to eliminate off-target effects. Using more than one TNBC model and assessing cell lines that represent other breast cancer subtypes will strengthen the findings and broaden the conclusion reported in this study. The primary focus of the current study was to assess the effect of fascin expression on the metabolomics profile of the MDA-MB-231 breast cancer cell line in order to identify potential candidates for further investigation and hypothesis generation. To guide subsequent targeted research, rather than making definitive conclusions, we consciously decided to refrain from stringent FDR correction, which requires larger datasets. The use of NP-G2-044 showed promising results in preclinical studies, where it inhibited metastasis and increased the overall survival of not only breast cancer-bearing mice, but also mice bearing several tumor [47,48]. These findings should encourage further studies to extend our results in vivo using cell line or patient-derived xenograft tumor models as well as to assess fascin’s function in the tumor microenvironment. Evaluating the effect of manipulating some of the dysregulated metabolites on breast cancer cell proliferation, migration and invasion in the context of fascin expression would strengthen the link between fascin-mediated dysregulation of these metabolites and the observed functions. Fascin has been shown by our group and others to regulate many pathways and downstream targets that are known to promote disease progression (reviewed in [20]). Therefore, it is intriguing to explore the effect of fascin-mediated metabolite dysregulation on these pathways and downstream targets in samples from breast cancer patients. Investigating fascin functions beyond its effect on metabolism and correlating the levels of dysregulated metabolites with fascin expression in samples from breast cancer patients will indicate the clinical relevance of the findings reported in this study. Despite these limitations, dysregulation of metabolic activity is one of the main mechanisms that cancer cells use to sustain their proliferation and thus has been therapeutically exploited. The outcome of this study led to a deeper understanding of fascin function, especially in influencing metabolic rewiring, a process that may alter cell metabolism and promote tumor growth. In the future, metabolic profiling may provide an alternative approach that can be exploited solo or in combination with selected histopathological markers for early noninvasive diagnosis and/or precise prediction of the disease progression or treatment outcome.

## 4. Materials and Methods

### 4.1. Cells and Reagents

The MDA-MB-231 (HTB-26) breast cancer cell lines were purchased from ATCC (Manassas, VA, USA). Cells were maintained in complete media of DMEM containing 10% fetal bovine serum (Invitrogen, Paisley, UK), 200 mM L-glutamine (Invitrogen) and antibiotic-antimycotic liquid (Invitrogen) at 37 °C in a 5% CO_2_ humidified atmosphere, and routinely screened for mycoplasma using a PCR-based kit from iNtRON (Seongnam-si, Republic of Korea), as previously described [19]. 

The primary monoclonal mouse anti-human fascin antibody was from DAKO (Santa Clara, CA, USA), and the secondary goat anti-mouse IgG1 antibody (APC) was purchased from Jackson ImmunoResearch Labs (West Grove, PA, USA). Both were used for fascin detection on flow cytometry.

### 4.2. Gene Knockdown and Restoration

MDA-MB-231 breast cancer cells are naturally fascin-positive. The establishment of stable fascin knockdown (fascin^KD^) and control (fascin^pos^) in MDA-MB-231 cells using fascin and scrambled shRNA, respectively, was previously described [14]. Furthermore, the rescue of fascin expression in the fascin^KD^ MDA-MB-231 cells using fascin ORF (fascin^KD+FORF^) was also previously described [19]. For transfection control, empty ORF was used in fascin^KD^ (fascin^KD+NORF^) and fascin^pos^ (fascin^pos+NORF^) cells. Fascin expression or knockdown was routinely checked at the RNA and protein levels.

### 4.3. RT-qPCR and Flow Cytometry

Quantitative Real-Time PCR (RT-qPCR) was carried out as previously described [14] using TaqMan fluorogenic probes (Applied Biosystems; Thermo Fisher Scientific, Waltham, MA, USA) on Applied Biosystems 7500 Fast detection system. RT-qPCR results were analyzed as previously described, and the data were presented as mean of triplicates ± SD of three independent experiments.

Fascin staining, detection, and analysis on flow cytometry (LSR II; Becton Dickinson, Mountain View, CA, USA) were performed as previously described [14].

### 4.4. Functional Assays

As previously described [49], the tumorsphere assay was performed and counted (cutoff size of 50 µ) using EVOS digital inverted microscope by Thermo Fisher Scientific (Waltham, MA, USA). For the colony-forming assay, the colonies were fixed, stained with crystal violet, and counted (blue dots) as previously described [49]. 

Live cell migration was assessed using CIM-plate 16 on xCELLigence Real Time Cell Analysis (RTCA) instrument, from ACEA Biosciences Inc. (San Diego, CA, USA) as previously described [16]. Data acquisition and analysis was carried out with the xCELLigence software (RTCA software 2.0.0.1301), which calculates the average migration of all the replicates by increases in the slope (1/h) of the curve during 24 h.

### 4.5. Sample Preparation

Equal numbers of the different groups of cells were seeded in complete media at 1 × 106/well in a 6-well plate. The complete media were then removed and the cells were then incubated in serum-free media for 24 h. The cell-free supernatants (secretomes) were first collected into labelled tubes. The plates containing the remaining cells (cell pellets) were washed with chilled 1x cold PBS and dipped in liquid nitrogen to quench the metabolism and reduce the experimental variations to extract the metabolites as previously described [50]. Briefly, 1 mL of cold 80% methanol: water was added to each plate for metabolite extraction, and cells were detached using a cell scraper and transferred to 1.5 mL Eppendorf tubes. The mixtures were vortexed at 4 °C, 600 rpm for 1 h in Thermomixer (Eppendorf, Germany). The samples were spun down at 4 °C, 16,000 rpm for 10 min (Eppendorf, Germany). 

Similarly, 900 µL of extraction solvent 1:1 (*v*/*v*) acetonitrile: methanol (ACN: MeOH) was added to 100 µL of the above collected supernatants for secretome metabolite extraction. The mixtures were vortexed in Thermomixer (Eppendorf, Germany) at 600 rpm, 4 °C for 1 h. The samples were spun down at 16,000 rpm, 4 °C for 10 min (Eppendorf, Germany), and then the secretomes were transferred to new Eppendorf tubes. 

The cell pellets and secretome extracts were completely evaporated in a SpeedVac (Christ, Germany) and stored at −80 °C until LC-MS analysis [51,52].

### 4.6. LC-MS Metabolomics

The dried extract samples were reconstituted in a 1:1 mobile phase (A: 0.1% formic acid in dH_2_O and B: 0.1% formic acid in (1:1) (*v*/*v*) MeOH: ACN) for an LC-MS metabolomics analysis [50]. First, 5 µL of the sample was introduced to the inlet technique, where the metabolites were separated in reversed-phase liquid chromatography using an ACQUITY UPLC XSelect (100 × 2.1 mm × 2.5 μm) column (Waters Ltd., Elstree, UK). The mobile phase flow rate was set at 300 μL/min, the column temperature was maintained at 55 °C, and the samples were maintained at 4 °C in the autosampler. Mobile phases A and B were pumped to the column in a gradient mode (0–16 min 95–5% A, 16–19 min 5% A, 19–20 min 5–95% A, and 20–22 min 5–95% A). The molecules eluted from the LC were positively or negatively ionized using an electrospray ionization source (ESI) and separated in the gas phase based on *m*/*z* using a Xevo G2-S QTOF mass spectrometer (Waters Ltd., Elstree, UK). The metabolites were ionized in the ESI source, where the source temperature was 150 °C, the desolvation temperature was 500 °C, and the capillary voltages were kept at 3.20 kV (ESI+) or 3 kV (ESI−). The cone voltage was 40 V. The desolvation gas flow was 800.0 L/h, and the cone gas flow was 50 L/h. The collision energies of the low and high functions were set to off and 10–50 V, respectively, in the MSE data-independent acquisition (DIA) mode. The mass spectrometer was calibrated, as recommended by the vendor, with sodium formate in the range of 100–1200 Da in both ionization modes. The lock mass compound, leucine-enkephaline (an external reference to the ion *m*/*z* 556.2771 in (ESI+) and 554.2615 (ESI−)), was injected continuously, switching between the sample and the reference every 45 and 60 s for ESI+ and ESI−, respectively, for a 0.5 s scan time, a flow rate of 10 µL/min, a cone voltage of 30 V, and a collision energy of 4 V. DIA were collected in continuum mode with a Masslynx™ V4.1 workstation (Waters Inc., Milford, MA, USA). Quality control samples (QCs) were collected by pooling 10 µL from each study sample and extracted. The QCs were then introduced into the instrument randomly, every 5th sample, to validate the system’s stability and were analyzed following the routine protocol.

### 4.7. Data and Statistical Analyses

The MS raw data were processed following a standard pipeline starting from alignment based on retention time and mass-to-charge ratio (*m/z*) correction followed by the compound peak picking. Compound signals were selected based on the quality of the peaks that had been selected, where the noise peaks were excluded outside the RT range of 0.5–20 min, and fragmentation ions with intensity below 0.2% using the Progenesis QI v.3.0 software from Waters (Waters Technologies, Milford, MA, USA).

Multivariate statistical analysis was performed using MetaboAnalyst version 5.0 (McGill University, Montreal, QC, Canada) (http://www.metaboanalyst.ca) (accessed on 21 April 2023) [53] For proper selection, the right statistical model and the datasets (compounds and raw abundances) were normalized by median, Pareto-scaled, and log-transformed to maintain their normal distribution. The normalized datasets generated partial least squares–discriminant analysis (PLS-DA) and orthogonal partial least squares–discriminant analysis (OPLS-DA) models. The OPLS-DA models created were evaluated using the fitness of the model (R2Y) and predictive ability (Q2) values [54]. Pathway analysis was performed on endogenous metabolites to identify the most affected pathway.

Univariate analysis was performed using Mass Profiler Professional v.15 (MPP) software (Agilent, Santa Clara, CA, USA). A volcano plot was used to identify significantly altered mass features based on Moderated *t*-test, *p*-value ≤ 0.05, and fold change cut-off 1.5. Venn diagrams were developed using MPP Software v15.0 (Agilent Inc., Santa Clara, CA, USA) [55].

### 4.8. Metabolite Identification

The significant features in each dataset were selected using Progenesis QI v.3.0 software for peak annotation. The chemical structures of metabolites were identified by acquiring their accurate precursor masses, fragmentation patterns, and isotopic distribution. They were set to a 5 ppm mass window for the Human Metabolome Database (HMDB) [56] and 5 ppm for METLIN MS/MS (https://metlin.scripps.edu/landing_page.php?pgcontent=mainPage), accessed 20 January 2024, using fragmentations filtered by in silico or empirical, KEGG, Lipid map, and Lipid Blast. The exogenous compounds, such as drugs, food additives, and environmental compounds, were excluded from the final list.

## Data Availability

The raw data of this study were deposited at Metabolomics Workbench and can be accessed under accession number ST003305 from 30 July 2024.

## Supplementary Materials

The following supporting information can be downloaded at: https://www.mdpi.com/article/10.3390/ijms25147891/s1.
