# Supplementary material for: Comparative Analysis of Breast Cancer Metabolomes Highlights Fascin’s Central Role in Regulating Key Pathways Related to Disease Progression
Source: Int J Mol Sci. 2024 Jul 18;25(14):7891. doi: 10.3390/ijms25147891 (PMC11277536; doi:10.3390/ijms25147891)
Supplement: Supplementary file 1 [file ijms-25-07891-s001.zip › Supplementary Figures (S1-S9).pptx]

## Slide 1
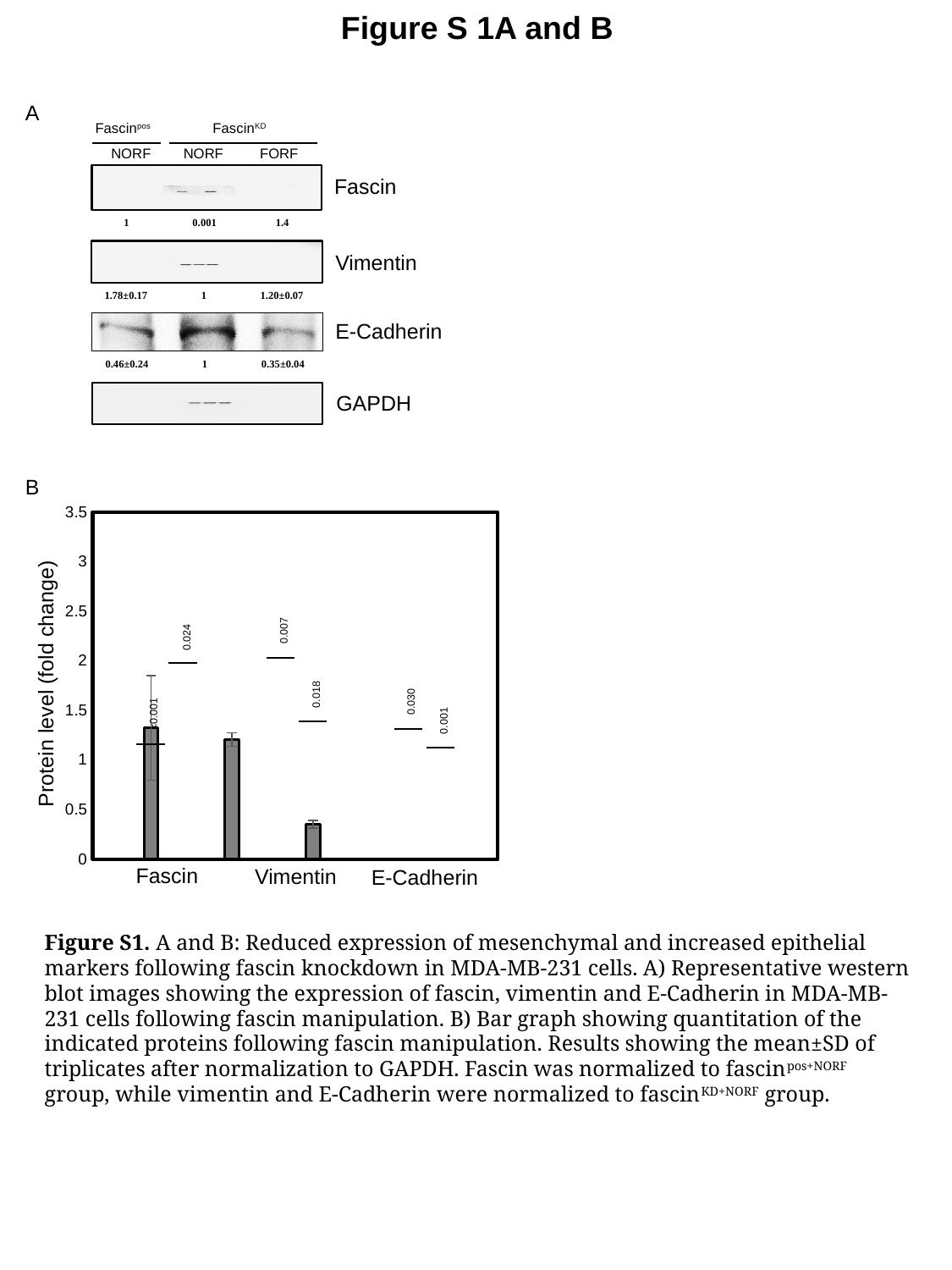

Figure S 1A and B
A
FascinKD
Fascinpos
NORF
NORF
FORF
Fascin
| 1 | 0.001 | 1.4 |
| --- | --- | --- |
Vimentin
| 1.78±0.17 | 1 | 1.20±0.07 |
| --- | --- | --- |
E-Cadherin
| 0.46±0.24 | 1 | 0.35±0.04 |
| --- | --- | --- |
GAPDH
B
### Chart
| Category | | | |
|---|---|---|---|0.007
0.024
0.018
0.030
0.001
0.001
Fascin
Vimentin
E-Cadherin
Protein level (fold change)
Figure S1. A and B: Reduced expression of mesenchymal and increased epithelial markers following fascin knockdown in MDA-MB-231 cells. A) Representative western blot images showing the expression of fascin, vimentin and E-Cadherin in MDA-MB-231 cells following fascin manipulation. B) Bar graph showing quantitation of the indicated proteins following fascin manipulation. Results showing the mean±SD of triplicates after normalization to GAPDH. Fascin was normalized to fascinpos+NORF group, while vimentin and E-Cadherin were normalized to fascinKD+NORF group.

## Slide 2
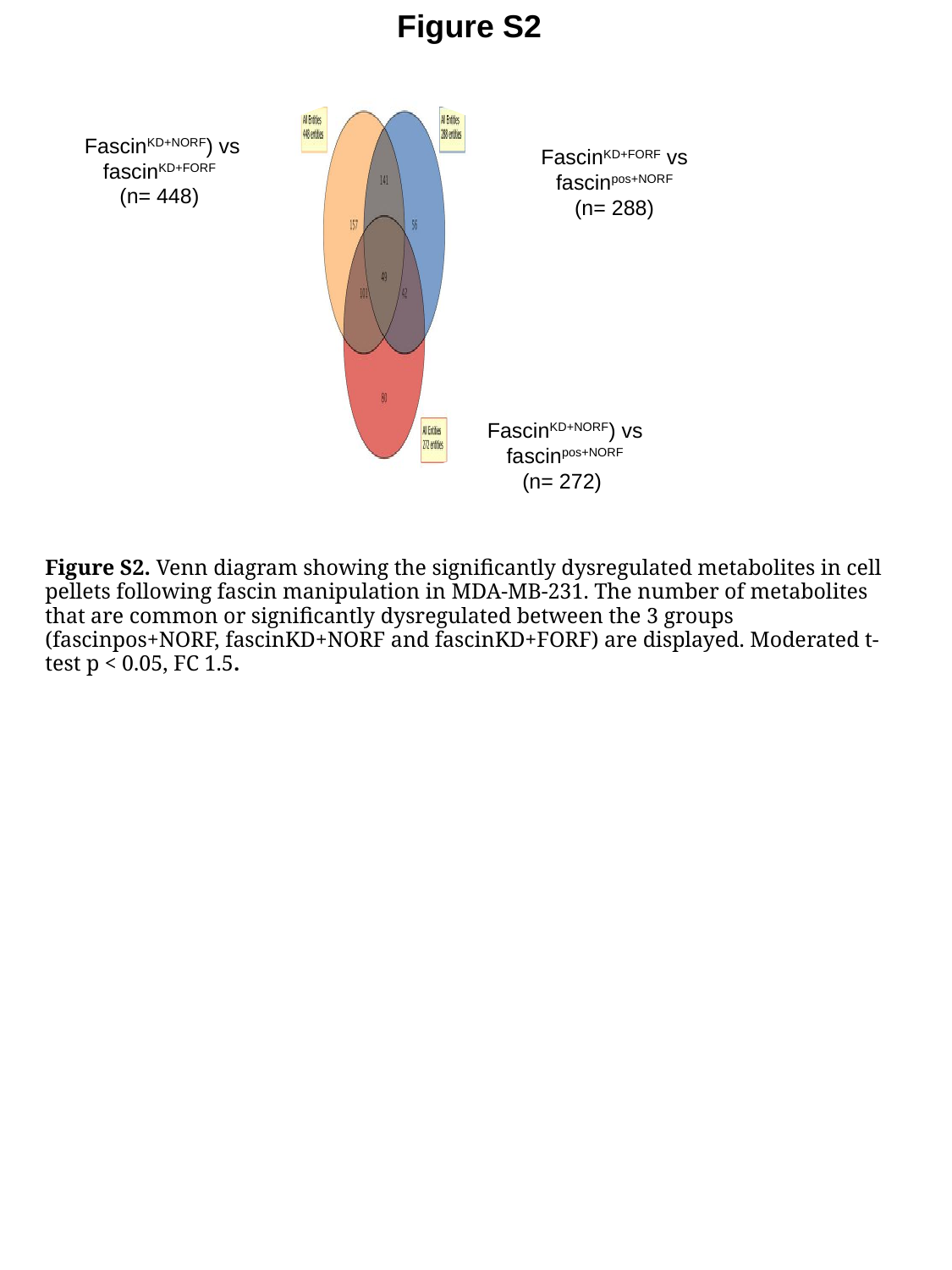

Figure S2
FascinKD+NORF) vs fascinKD+FORF
(n= 448)
FascinKD+FORF vs fascinpos+NORF
(n= 288)
FascinKD+NORF) vs fascinpos+NORF
(n= 272)
Figure S2. Venn diagram showing the significantly dysregulated metabolites in cell pellets following fascin manipulation in MDA-MB-231. The number of metabolites that are common or significantly dysregulated between the 3 groups (fascinpos+NORF, fascinKD+NORF and fascinKD+FORF) are displayed. Moderated t-test p < 0.05, FC 1.5.

## Slide 3
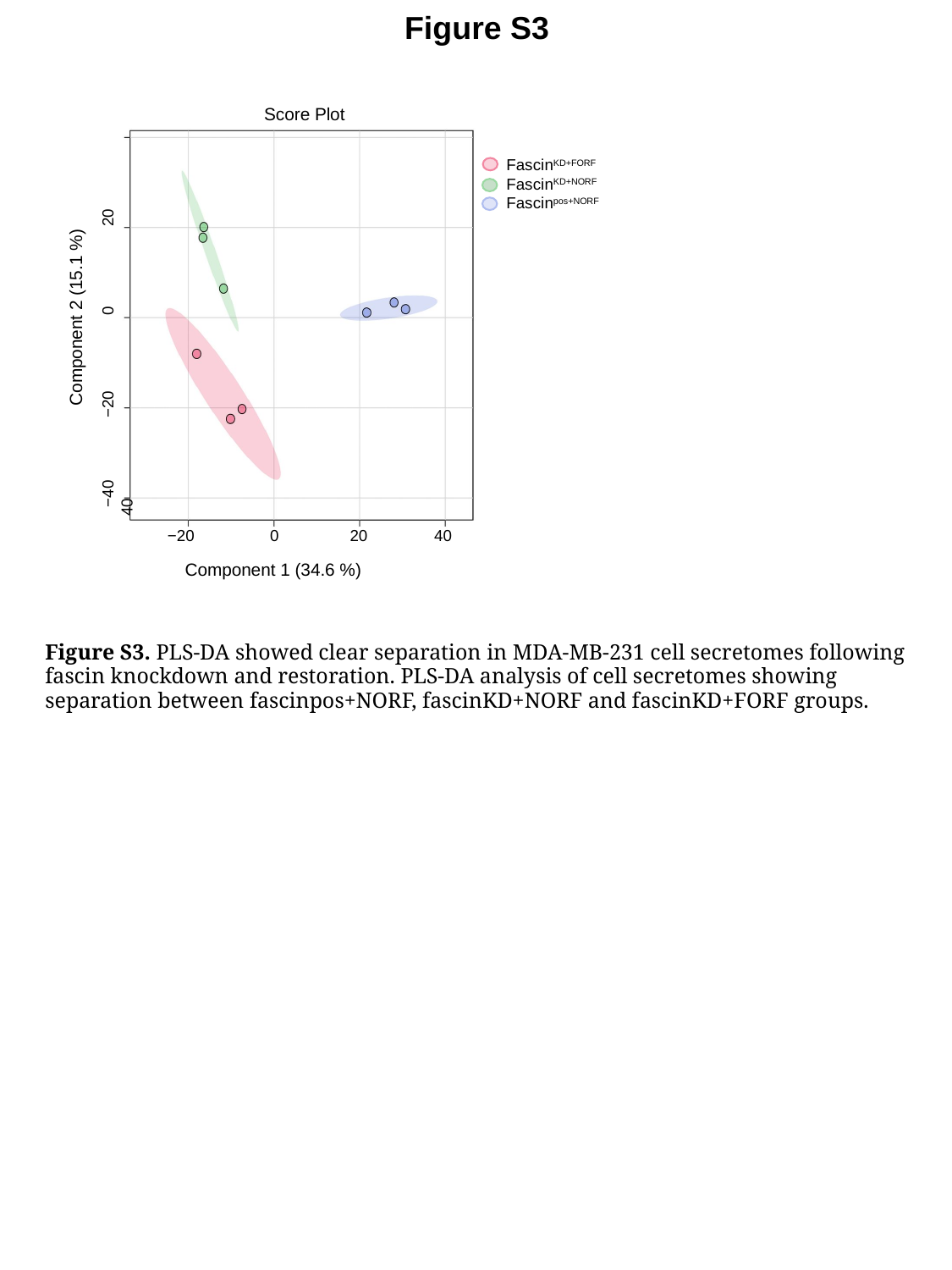

Figure S3
Score Plot
 −40 −20 0 20 40
Component 2 (15.1 %)
−20 0 20 40
Component 1 (34.6 %)
FascinKD+FORF
FascinKD+NORF
Fascinpos+NORF
Figure S3. PLS-DA showed clear separation in MDA-MB-231 cell secretomes following fascin knockdown and restoration. PLS-DA analysis of cell secretomes showing separation between fascinpos+NORF, fascinKD+NORF and fascinKD+FORF groups.

## Slide 4
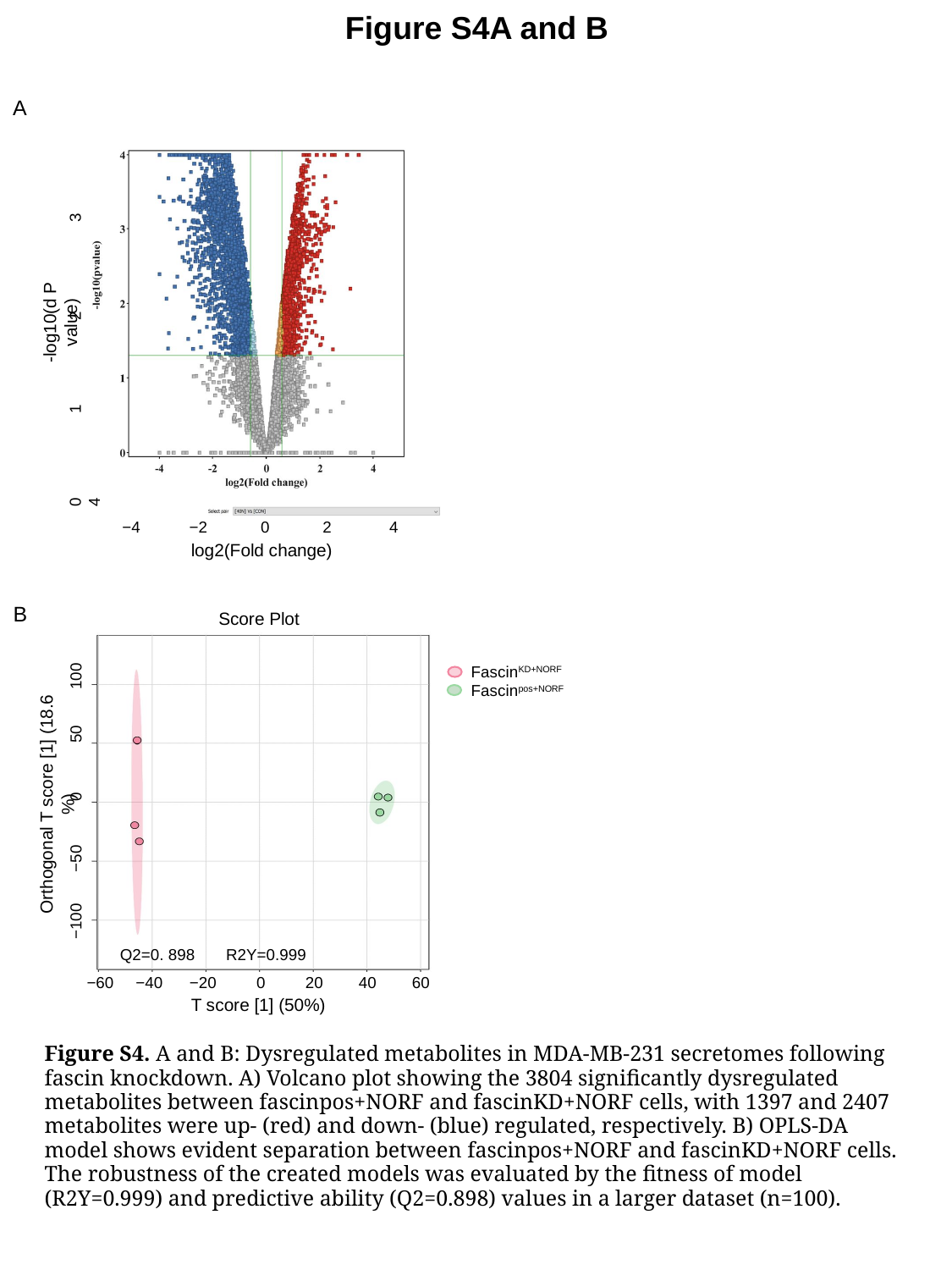

Figure S4A and B
A
 0 1 2 3 4
-log10(d P value)
 −4 −2 0 2 4
log2(Fold change)
B
Score Plot
−100 −50 0 50 100
Orthogonal T score [1] (18.6 %)
Q2=0. 898 R2Y=0.999
−60 −40 −20 0 20 40 60
T score [1] (50%)
FascinKD+NORF
Fascinpos+NORF
Figure S4. A and B: Dysregulated metabolites in MDA-MB-231 secretomes following fascin knockdown. A) Volcano plot showing the 3804 significantly dysregulated metabolites between fascinpos+NORF and fascinKD+NORF cells, with 1397 and 2407 metabolites were up- (red) and down- (blue) regulated, respectively. B) OPLS-DA model shows evident separation between fascinpos+NORF and fascinKD+NORF cells. The robustness of the created models was evaluated by the fitness of model (R2Y=0.999) and predictive ability (Q2=0.898) values in a larger dataset (n=100).

## Slide 5
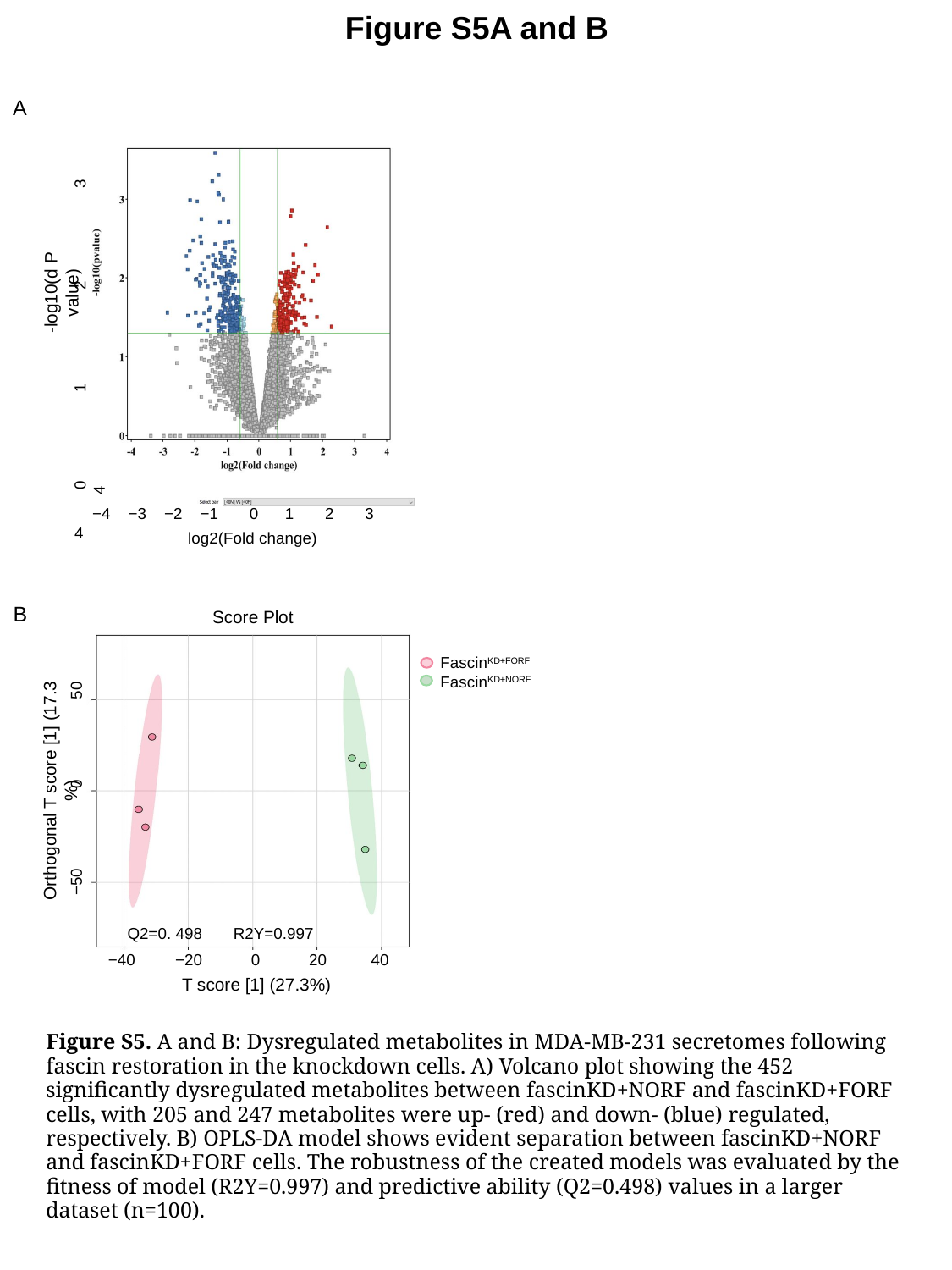

Figure S5A and B
A
 0 1 2 3 4
-log10(d P value)
 −4 −3 −2 −1 0 1 2 3 4
log2(Fold change)
B
Score Plot
−50 0 50
Orthogonal T score [1] (17.3 %)
Q2=0. 498 R2Y=0.997
−40 −20 0 20 40
T score [1] (27.3%)
FascinKD+FORF
FascinKD+NORF
Figure S5. A and B: Dysregulated metabolites in MDA-MB-231 secretomes following fascin restoration in the knockdown cells. A) Volcano plot showing the 452 significantly dysregulated metabolites between fascinKD+NORF and fascinKD+FORF cells, with 205 and 247 metabolites were up- (red) and down- (blue) regulated, respectively. B) OPLS-DA model shows evident separation between fascinKD+NORF and fascinKD+FORF cells. The robustness of the created models was evaluated by the fitness of model (R2Y=0.997) and predictive ability (Q2=0.498) values in a larger dataset (n=100).

## Slide 6
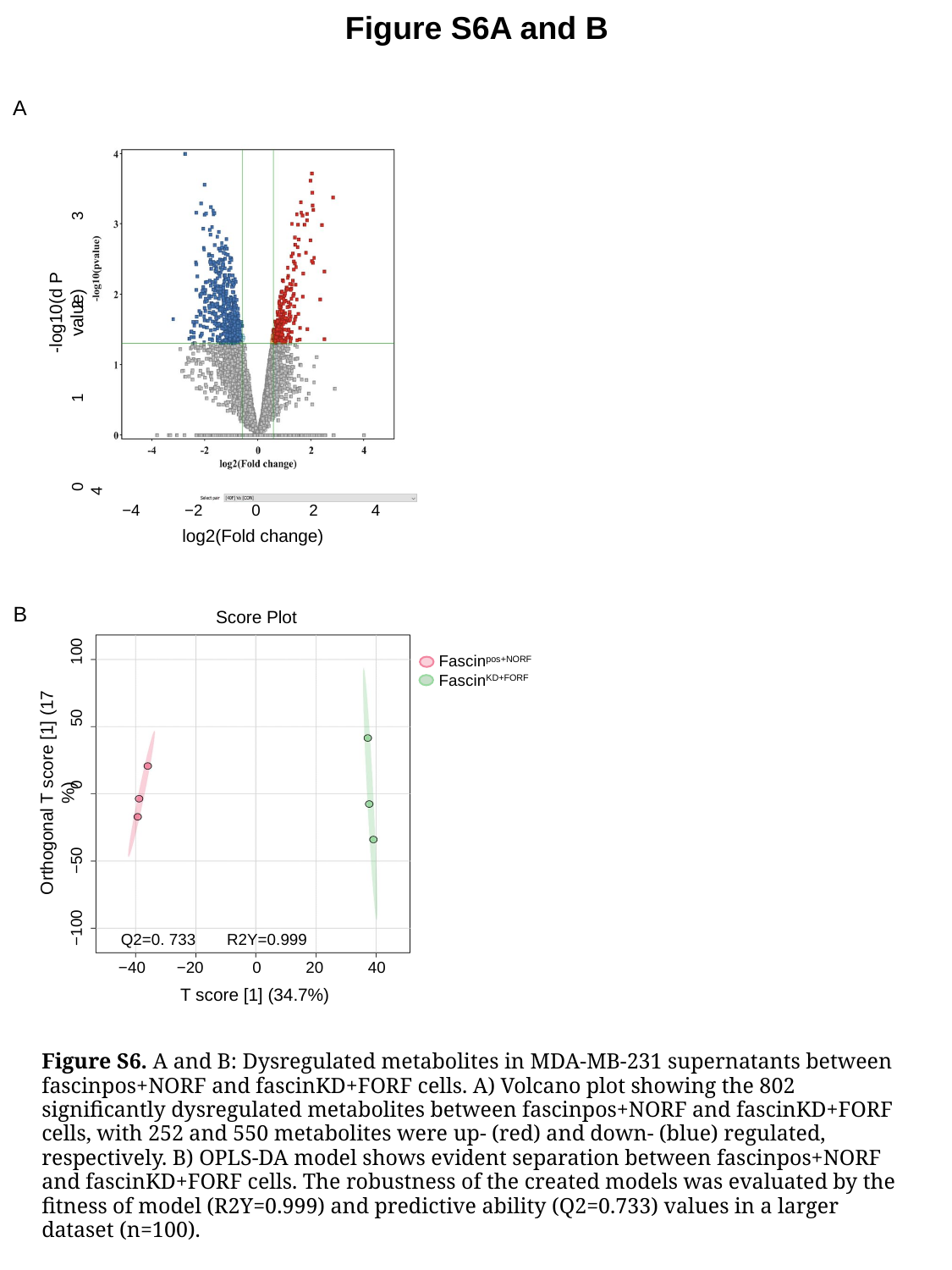

Figure S6A and B
A
 0 1 2 3 4
-log10(d P value)
 −4 −2 0 2 4
log2(Fold change)
B
Score Plot
−100 −50 0 50 100
Orthogonal T score [1] (17 %)
 −40 −20 0 20 40
T score [1] (34.7%)
Q2=0. 733 R2Y=0.999
Fascinpos+NORF
FascinKD+FORF
Figure S6. A and B: Dysregulated metabolites in MDA-MB-231 supernatants between fascinpos+NORF and fascinKD+FORF cells. A) Volcano plot showing the 802 significantly dysregulated metabolites between fascinpos+NORF and fascinKD+FORF cells, with 252 and 550 metabolites were up- (red) and down- (blue) regulated, respectively. B) OPLS-DA model shows evident separation between fascinpos+NORF and fascinKD+FORF cells. The robustness of the created models was evaluated by the fitness of model (R2Y=0.999) and predictive ability (Q2=0.733) values in a larger dataset (n=100).

## Slide 7
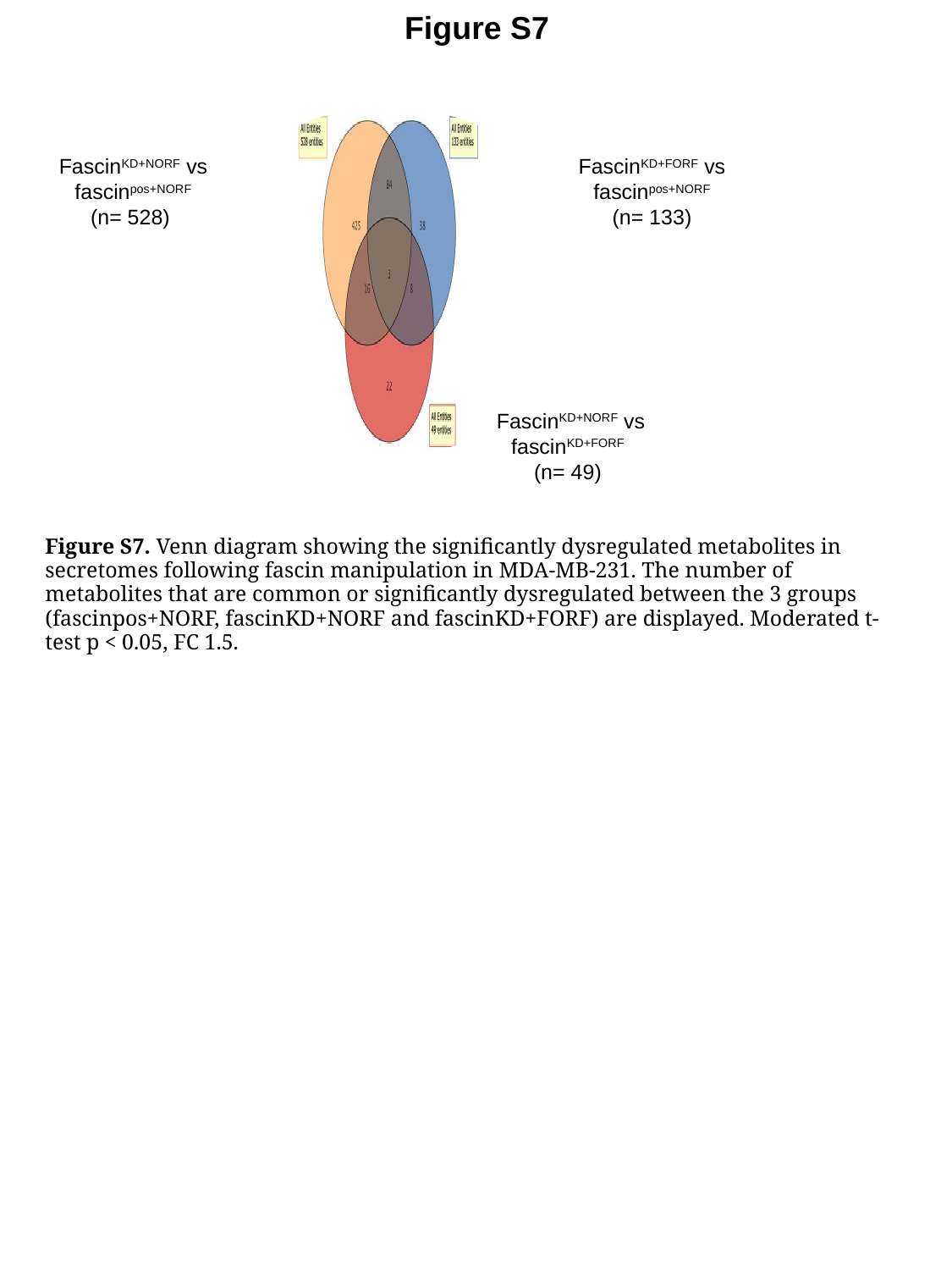

Figure S7
FascinKD+NORF vs fascinpos+NORF
(n= 528)
FascinKD+FORF vs fascinpos+NORF
(n= 133)
FascinKD+NORF vs fascinKD+FORF
(n= 49)
Figure S7. Venn diagram showing the significantly dysregulated metabolites in secretomes following fascin manipulation in MDA-MB-231. The number of metabolites that are common or significantly dysregulated between the 3 groups (fascinpos+NORF, fascinKD+NORF and fascinKD+FORF) are displayed. Moderated t-test p < 0.05, FC 1.5.

## Slide 8
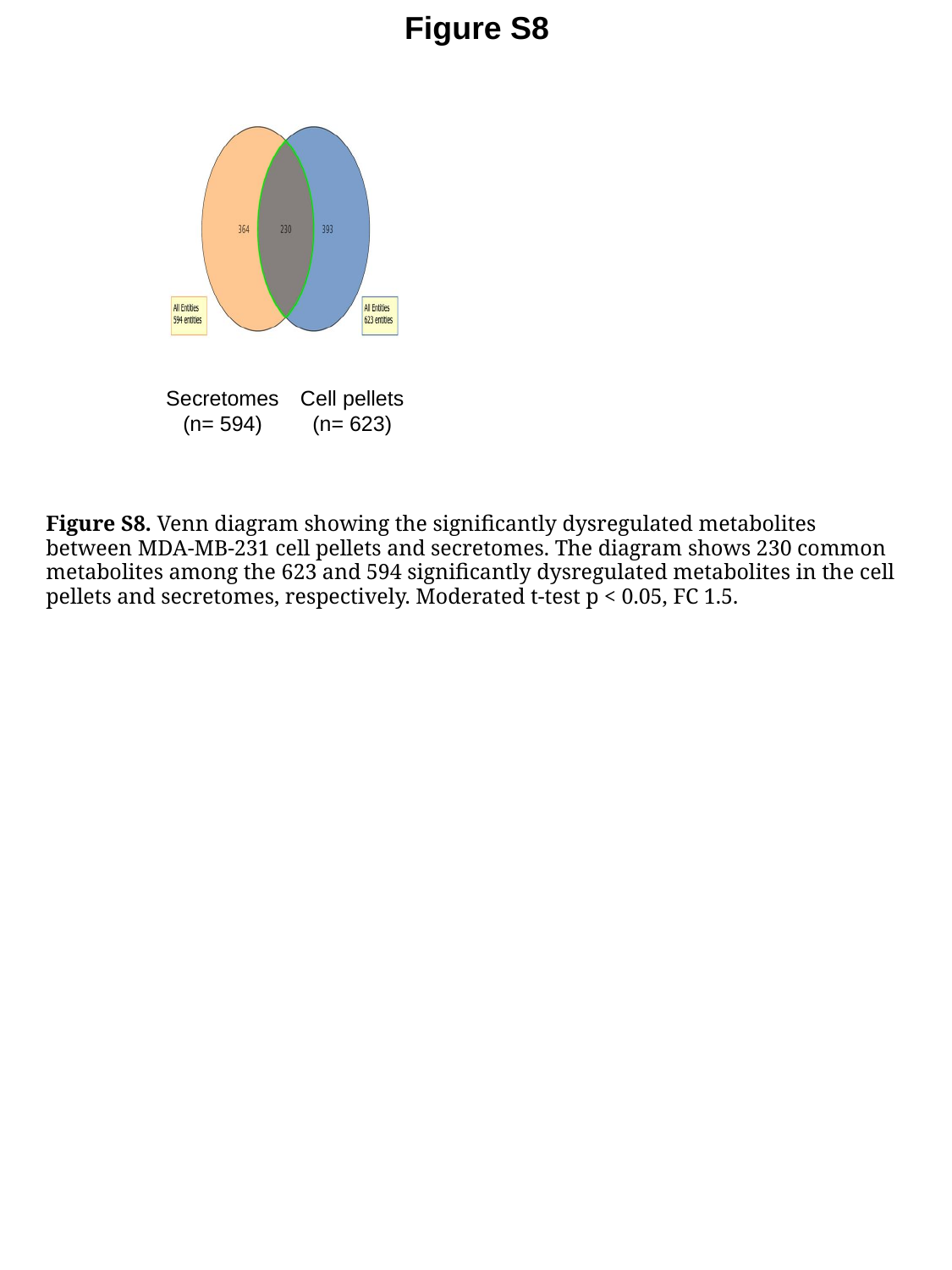

Figure S8
 Cell pellets
(n= 623)
Secretomes
(n= 594)
Figure S8. Venn diagram showing the significantly dysregulated metabolites between MDA-MB-231 cell pellets and secretomes. The diagram shows 230 common metabolites among the 623 and 594 significantly dysregulated metabolites in the cell pellets and secretomes, respectively. Moderated t-test p < 0.05, FC 1.5.

## Slide 9
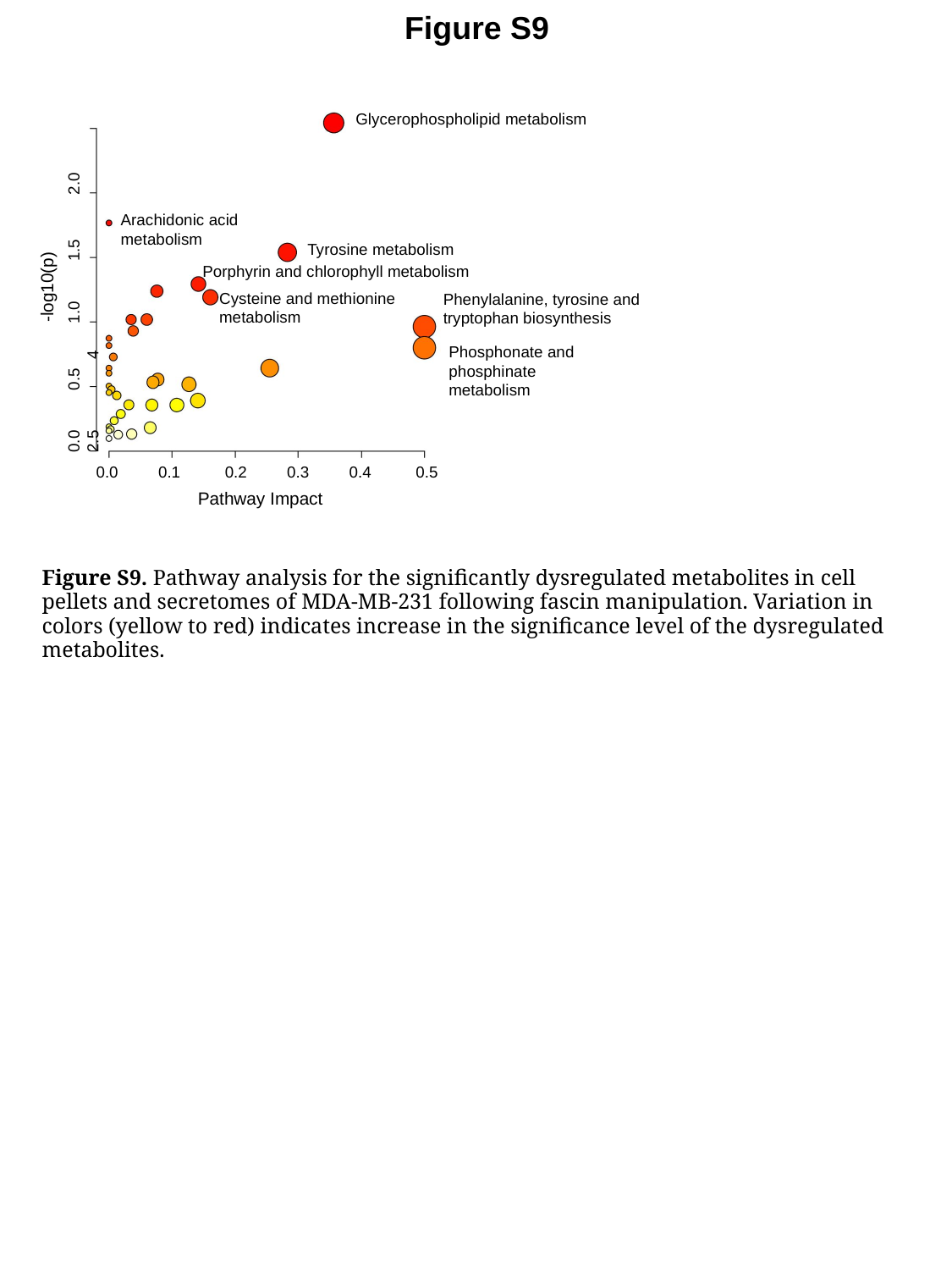

Figure S9
Glycerophospholipid metabolism
Arachidonic acid metabolism
Tyrosine metabolism
Porphyrin and chlorophyll metabolism
-log10(p)
Cysteine and methionine metabolism
Phenylalanine, tyrosine and tryptophan biosynthesis
Phosphonate and phosphinate
metabolism
Pathway Impact
0.0 0.5 1.0 1.5 2.0 2.5 4
0.0 0.1 0.2 0.3 0.4 0.5
Figure S9. Pathway analysis for the significantly dysregulated metabolites in cell pellets and secretomes of MDA-MB-231 following fascin manipulation. Variation in colors (yellow to red) indicates increase in the significance level of the dysregulated metabolites.
